# Latest Progress in Risk-Adapted Surgery for Medullary Thyroid Cancer

**DOI:** 10.3390/cancers16050917

**Published:** 2024-02-24

**Authors:** Andreas Machens, Kerstin Lorenz, Tim Brandenburg, Dagmar Führer, Frank Weber, Henning Dralle

**Affiliations:** 1 Department of Visceral, Vascular and Endocrine Surgery, Martin Luther University Halle-Wittenberg, Ernst-Grube-Str. 40, D-06097 Halle (Saale), Germany; kerstin.lorenz@uk-halle.de; 2 Department of Endocrinology, Diabetology and Metabolism, University of Duisburg-Essen, Hufelandstraße 55, D-45147 Essen, Germany; tim.brandenburg@uk-essen.de (T.B.); dagmar.fuehrer-sakel@uk-essen.de (D.F.); 3 Department of General, Visceral and Transplantation Surgery, Division of Endocrine Surgery, University of Duisburg-Essen, Hufelandstraße 55, D-45147 Essen, Germany; frank.weber@uk-essen.de (F.W.); henning.dralle@uk-essen.de (H.D.)

**Keywords:** medullary thyroid cancer, ultrasound, frozen section, desmoplasia, risk-adapted surgery, ipsilateral central node dissection, surgical morbidity, biochemical cure

## Abstract

**Simple Summary:**

Medullary thyroid cancer is a rare neuroendocrine tumor, which is inherited in 25% of cases. Medical progress has made it easier to find thyroid tumors before they spread to the neck nodes, which makes a surgical cure harder to achieve. Removing only the tumor-bearing thyroid lobe causes fewer surgical complications than removing the whole thyroid gland or neck nodes. When the remaining thyroid lobe produces enough thyroid hormone, patients are spared the need to take one thyroid hormone tablet daily for life. This literature review confirms that the removal of the tumor-bearing thyroid lobe alone clears all of the tumor in patients who are not gene carriers and whose thyroid tumors showed no ‘desmoplasia’ under the microscope on a rapid ‘frozen section’ examination. Gene carriers, who can be detected by a simple blood test, still need to have the whole thyroid gland removed so that no other thyroid tumors can form from the thyroid tissue left behind.

**Abstract:**

(1) Background: The wider adoption of a preoperative ultrasound and calcitonin screening complemented by an intraoperative frozen section has increased the number of patients with occult sporadic medullary thyroid cancer (MTC). These advances offer new opportunities to reduce the extent of the initial operations, minimizing operative morbidity and the risk of postoperative thyroxin supplementation without compromising the cure. (2) Methods: This systematic review of the international literature published in the English language provides a comprehensive update on the latest progress made in the risk-adapted surgery for sporadic and hereditary MTC guided by an intraoperative frozen section. (3) Results: The current evidence confirms the viability of a hemithyroidectomy for desmoplasia-negative sporadic MTC. To add an extra safety margin, the hemithyroidectomy may be complemented by a diagnostic ipsilateral central node dissection. Despite the limited extent of the surgery, all the patients with desmoplasia-negative sporadic tumors achieved a biochemical cure with excellent clinical outcomes. A hemithyroidectomy decreases the need for postoperative thyroxine substitution, but a total thyroidectomy may be required for bilateral nodular thyroid disease. Hereditary MTC is a different issue. Because each residual thyroid C cell carries its own risk of malignant progression, a total thyroidectomy remains mandatory for hereditary MTC. (4) Conclusion: In experienced hands, a hemithyroidectomy, which minimizes morbidity without compromising the cure, is an adequate therapy for desmoplasia-negative sporadic MTC.

## 1. Introduction

Technological progress continues to transform the practice of medicine. In endocrine oncology, the adoption of high-resolution imaging, flanked by biochemical screening using increasingly more sensitive calcitonin assays, has expanded the pool of patients operated on for occult sporadic medullary thyroid cancer (MTC) [1,2,3]. This achievement is noteworthy because MTC, a slow-growing, calcitonin-secreting neuroendocrine malignancy, has a proclivity for early spread to locoregional neck nodes and beyond [4].

Achieving a biochemical cure is the foremost goal of neck surgery, which is the single most effective treatment modality for MTC. Establishing a diagnosis of cancer before the operation leads to oncologically more adequate operations and more frequent cures [5,6]. The basic premise underlying screening programs is that the identification of an asymptomatic subclinical disease catches tumors early in their growth trajectory, before they can spread to the neck nodes and distant organs and cause clinical signs and symptoms [7]. Owing to their frequent miliary growth, the neck nodes may be too small for fine-needle aspiration, evading detection by even the most sophisticated imaging modalities. Depending on their number and location, node metastases make it harder or impossible to reach a biochemical cure, even in experienced hands [8].

In the past, the clinical work-up for thyroid cancer used to hinge on neck palpation. Since the 1990s, though, palpation has largely yielded to high-resolution neck ultrasound, which picks up smaller and deep-seated thyroid tumors better than neck palpation, but is not perfect either. In parallel, intraoperative frozen section is increasingly being utilized in the operating suite. Frozen section is a powerful tool that distinguishes well (i) between tumors involving one vs. both thyroid lobes, and (ii) between tumors confined to the thyroid and tumors invading the neck. When this crucial information breaks in a timely fashion, it can give the surgeon valuable clues as to the extent of tumor spread and the required extent of neck surgery.

Conceptionally, frozen section analysis could be expanded to include reliable tissue biomarkers predictive of lymphatic spread. Indeed, a number of histopathological publications, which appeared in the 2000s, pointed to a clinically useful link between a desmoplastic stromal reaction, referred to as desmoplasia in this review article, and node metastases in MTC [9,10,11]. Desmoplasia is defined as the presence of newly formed fibrotic (collagenous) stroma surrounding the invasive epithelial tumor cells, which is not seen in non-neoplastic thyroid parenchyma [11]. In the late 2010s and early 2020s, these initial reports were confirmed [12] and extended by independent series that defined the risk-reducing corridors of opportunity for desmoplasia-negative sporadic MTC without compromising the cure [13]. The absence of desmoplasia obviates the need for a total thyroidectomy and central node dissection for sporadic MTC, decreasing morbidity due to the compartment-oriented surgery and the need for postoperative thyroxine substitution [14].

Given the rapid evolution of precision surgery in the past ten years, it may be worthwhile to review what important information tumor desmoplasia holds for surgeons operating on patients with sporadic MTC. The present systematic review was undertaken to provide a comprehensive update on the latest progress made regarding targeted, risk-reducing surgery for medullary thyroid cancer.

## 2. Methods

### 2.1. Eligibility Criteria

Briefly, the inclusion criteria for this systematic review included all literature types in the English language that covered desmoplasia in association with MTC, with the exception of review articles.

### 2.2. Databases and Search Strategy

A systematic review of the international literature was performed in accordance with the PRISMA (Preferred Reporting Items for Systematic Reviews and Meta-Analyses) guidelines [15], and has not been registered.

An electronic Medline search of the international literature published in the English language was conducted with a cutoff of 15 January 2024 using the following search terms: ‘desmoplasia’, ‘desmoplastic stromal reaction’, ‘medullary thyroid cancer’, and ‘medullary thyroid carcinoma’. The terms of this literature search were identified in the title, abstract, or medical subject heading. The publications that fulfilled the inclusion criteria and addressed the respective topic were further evaluated following the PRISMA guidelines (https://www.mdpi.com/editorial_process#standards, last accessed on 15 January 2024). Extracted were the relevant data, such as the reference details (authors, title, journal, and year of publication), histopathologic data, and clinical outcome variables, including the biochemical cure and locoregional recurrence.

### 2.3. Evidence-Based Medicine Grading of the Retrieved Literature

The search results were independently evaluated by 2 reviewers (A.M. and H.D.). The references meeting the inclusion criteria were assigned their respective levels of evidence using a modification of Sackett’s classification [16], proposed by Heinrich et al. [17] (Table 1). The initial grading was developed by 2 researchers (A.M. and H.D.), and approved by the consensus of all the authors.

## 3. Results

### 3.1. Results of the Literature Search

**Figure 1:**
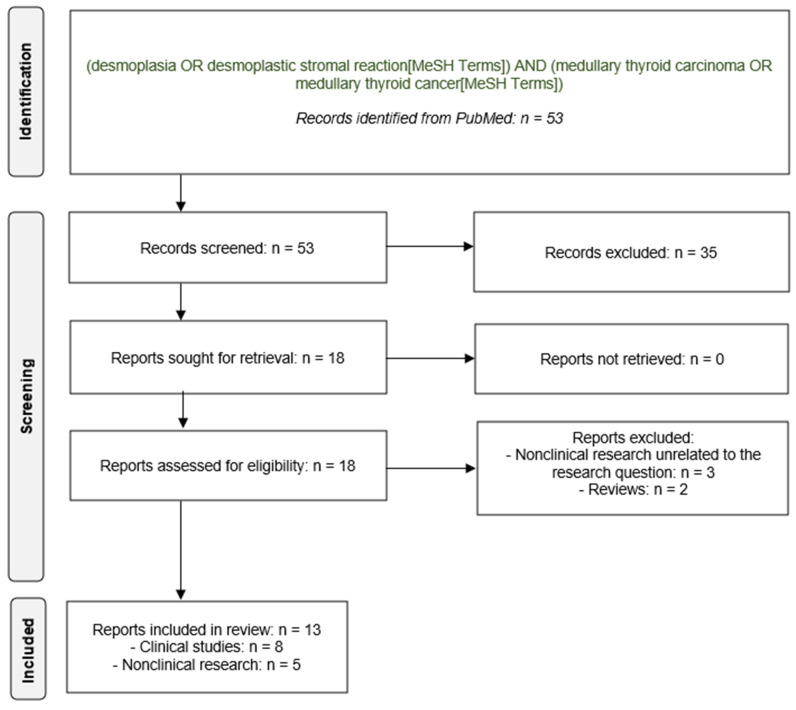
PRISMA flow diagram.

Figure 1 summarizes the flow of the records identified (53 reports), screened (53 reports), excluded (35 reports), retrieved (18 reports), assessed for eligibility (18 reports), and finally included in the review (13 reports): 13 publications, 8 were clinical investigations and 5 were nonclinical basic research reports, which the present systematic review will cover subsequently in more detail.

### 3.2. Experimental Data on Desmoplasia and Medullary Thyroid Cancer

For patients with MTC, there is a paucity of data explaining the association between primary tumor desmoplasia on the one hand, and larger primary thyroid tumors, tumor extension through the thyroid capsule (extrathyroid growth), the development of node metastases, and the failure to reach a biochemical cure, on the other. Laboratory research has shown that MTC cells consistently express fibroblast activation protein α, tenascin-C, hypoxia-induced factor 1α, and carbonic anhydrase IX [18,19]. Intriguingly, the expression of these tissue biomarkers agrees with the extent of the primary tumor desmoplasia and the presence of node metastases [18,19]. On the basis of these experimental findings, it has been hypothesized that desmoplasia may represent an attempt by the body to heal the tissue injury produced by the infiltrative and destructive growth of tumor cells [11].

It can be hypothesized that these tissue biomarkers mediate the loosening of intercellular tissue connections, so that the malignant cells can break free from the primary thyroid tumor to travel via the lymphatic and hematogenous systems to the capillary bed of organs with a microenvironment conducive to their growth and implantation [20]. Likewise, these tissue biomarkers may also facilitate the penetration of the thyroid and nodal capsules, giving rise to extrathyroid extension (breach of the thyroid capsule) and extranodal growth (breach of the lymph node capsule), respectively.

Evidence level V, Grade of recommendation: –

### 3.3. Primary Tumor Desmoplasia in Sporadic Medullary Thyroid Cancer

The key question from a clinical perspective is whether primary tumor desmoplasia is related more closely to the primary tumor size, which can be easily measured on the surgical thyroid specimen [21], or to node metastases [10,11,22,23].

This important point was clarified in a retrospective study of 139 patients with unifocal sporadic MTC [13]. In this detailed clinical–histopathological analysis, the extent of the primary tumor desmoplasia (0%, <1%, 1–10%, 11–20%, 21–32%, 33–50%, and >50%) correlated more closely with the number of node metastases (Spearman’s ρ = 0.606; *p* < 0.001) than with the primary tumor size (Spearman’s ρ = 0.267; *p* = 0.002). Low–moderate-to-high desmoplasia with fibrosis, making up >10% of the representative thyroid tumor sections, yielded excellent sensitivity and a negative predictive value (100%) for node metastases, with moderate specificity (57% and 48%) and a positive predictive value (50% and 46%). In hindsight, node dissection was not necessary in 55 (57%) patients who harbored desmoplasia-negative MTC. Finally, when the frozen sections were histopathologically compared with the matching paraffin-embedded thyroid tumor specimens, the concordance was 98% (53 of 54 pairs): one of seven upgrades changed the diagnosis to ‘desmoplasia present’, whereas one of three downgrades shifted the diagnosis of tumor capsule breach from ‘present’ to ‘absent’ [13].

This proof-of-concept study paved the way for risk-reducing surgery for desmoplasia-negative unifocal sporadic MTC.

Evidence level IV, Grade of recommendation: –

Most recently, a careful study of 56 patients with sporadic MTC who were pre-screened for non-metastatic disease by ultrasound found that desmoplasia-negative tumors without preoperative or intraoperative evidence of disease outside the thyroid gland were never multifocal (0 of 19 vs. 2 of 6 patients, or 0% vs. 33%; *p* = 0.050) or node-negative (0 of 19 vs. 6 of 6 patients, or 0% vs. 100%; medians of 0 vs. 3.5 node metastases; both *p* < 0.001). Unlike the patients with a total thyroidectomy, no patient who underwent a hemithyroidectomy received postoperative thyroid hormone replacement therapy [14].

These results demonstrate that a hemithyroidectomy with diagnostic ipsilateral central neck dissection can safely replace a total thyroidectomy with bilateral central node dissection when the primary thyroid tumor is clinically non-metastatic and desmoplasia-negative [14].

When this innovative study (Table 2, right panel) was contrasted with a previous retrospective clinical study not enriched with non-metastatic sporadic MTC (Table 2, left panel) [11], the benefits of ultrasonographic enrichment with clinically non-metastatic MTC (Table 2, right panel) became obvious: 32 (27%) of 120 patients without pre-screening vs. 19 (76%) of 25 patients with pre-screening. Despite the differences in study design, both studies, coming from different angles, agreed that desmoplasia-negative tumors are always confined to the thyroid and are node-negative, making them biochemically curable without the dissection of the neck nodes.

**Figure 2:**
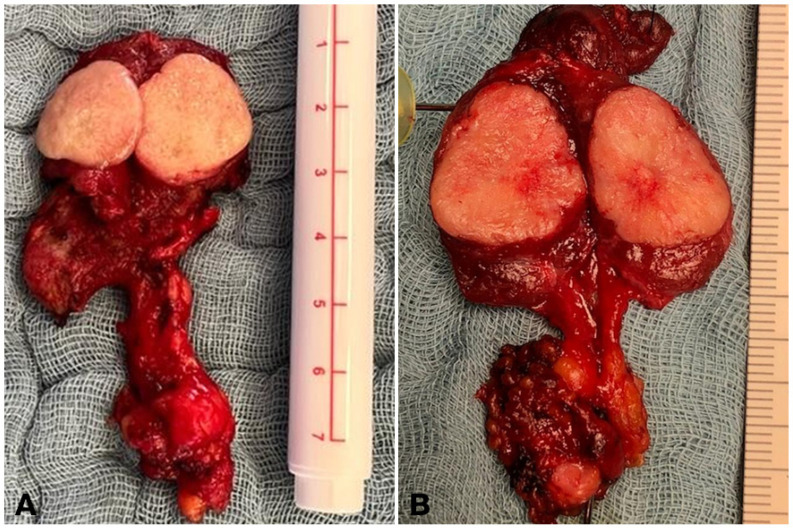
Intraoperative presentation of medullary thyroid cancer. (**A**). Surgical specimen of a desmoplasia-negative non-metastatic thyroid tumor with sharp, circumscribed margins. (**B**). Surgical specimen of a desmoplasia-positive, invasive, node-positive thyroid tumor with an inhomogeneous cut surface and blurred margins.

These desmoplasia-negative indolent tumors (Figure 2A), for which the term ‘Sporadic Noninvasive Medullary Thyroid Neoplasm (SNIMTN)’ has been proposed, need to be differentiated from invasive MTC (Figure 2B) [14].

Evidence level III, Grade of recommendation: C

### 3.4. Primary Tumor Desmoplasia in Hereditary Medullary Thyroid Cancer

Biochemical and genomic screening have moved the diagnosis of hereditary MTC forward in time. Many hereditary tumors identified by a family screening are fairly small and node-negative at diagnosis. In good keeping with this, a study of 11 patients with inherited medullary microcarcinomas observed lower incidences of stromal desmoplasia and node metastases compared to larger sporadic medullary carcinomas [9].

In contrast, another report on ≤10 mm large MTCs noted that hereditary MTC, which was characterized by multifocal growth (13 [81%] of 16 patients vs. 3 [9%] of 34 patients; *p* < 0.001), more often had histopathologic evidence of desmoplasia in the primary tumor than with sporadic MTC: 14 (87.5%) of 16 patients vs. 19 (56%) of 34 patients (*p* = 0.02) [10].

A recent study evaluated simultaneously the metastatic behavior of multiple primary thyroid tumors of disparate size and the extent of desmoplasia in three patients with hereditary MTC in the context of multiple endocrine neoplasia type 2B [24]. In this clinical–histopathological case series, the desmoplasia-negative 8, 11, and 16 mm large low-grade thyroid tumors with 5–7% desmoplasia did not spread to the ipsilateral neck nodes, whereas the desmoplasia-positive 6 mm small high-grade and the 7 mm small low-grade thyroid tumors with 35–40% desmoplasia did. Subject to confirmation by a larger series, the reported 10% threshold for primary thyroid desmoplasia for sporadic MTC [13] may extend to hereditary MTC as well [24].

To increase patient numbers, hereditary MTCs often are combined with sporadic MTCs, without a breakdown by RET risk category or RET mutation [10,25], or even without specifying the number of patients with hereditary or sporadic MTC [26].

Evidence level V, Grade of recommendation: –

For instance, one clinical study merged 93 RET carriers with hereditary MTC and 267 patients with sporadic MTC into one study group of 360 patients. Based on these combined data, the omission of lateral node dissection was advocated for patients with desmoplasia-negative MTC on an intraoperative frozen section [25]. Although none of the 64 (17.8%) desmoplasia-negative patients turned out to be node-positive, the authors stopped short of considering all forms of node dissection as an overtreatment for desmoplasia-negative MTC.

Evidence level III, Grade of recommendation: C

Within the limitations of the present data, there is some evidence to suggest that desmoplasia-negative hereditary MTCs do not biologically differ from desmoplasia-negative sporadic MTCs. To confirm this notion, additional clinical investigations, including larger numbers of RET carriers with hereditary MTCs, are required.

Evidence level V, Grade of recommendation: –

### 3.5. Nodal Desmoplasia in Medullary Thyroid Cancer

Nodal desmoplasia has rarely been studied in patients with MTC. A first, preliminary report on the subject found that the extent of nodal desmoplasia was greater in the presence of extranodal growth than with intranodal growth [24]. In this clinical series, nodal desmoplasia proved extremely useful for tracing patterns of lymphatic spread. Node metastases usually involve the ipsilateral central and lateral neck before crossing the midline to invade the contralateral central and lateral neck and, ultimately, the ipsilateral axilla [24]. Occasional exceptions include thyroid tumors in the upper thyroid lobe, which drain directly into the ipsilateral lateral neck via lymphatic channels that follow the superior thyroid vein [27,28]. These so-called ‘skip’ metastases directly invade the ipsilateral lateral neck while sparing the ipsilateral central neck [29].

These findings, subject to confirmation by a larger series, suggest that nodal desmoplasia may be a predictive tissue biomarker for hereditary MTC. Analogous to papillary thyroid microcarcinoma [30] and pancreatic cancer [31], nodal desmoplasia may be associated with extranodal growth and larger nodal size in MTC, and may predict tumor recurrence after a compartment-oriented neck dissection [24].

Evidence level V, Grade of recommendation: –

In contrast to hereditary MTC associated with multiple endocrine neoplasia 2B, there are no reports on nodal desmoplasia with sporadic MTC or hereditary MTC associated with multiple endocrine neoplasia 2A as of this writing, which warrant additional research.

## 4. Discussion

### 4.1. Key Results

The absence of primary tumor desmoplasia, a predictive tissue biomarker of nodal spread on an intraoperative frozen section and a definitive histopathology, reliably indicates the absence of node metastases in patients with sporadic MTC. Up to one-third (19 [34%] of 56 patients) of sporadic MTCs are desmoplasia-negative on the definitive histopathology and, hence, node-negative, as evidenced by the normalized postoperative calcitonin serum levels (Table 2, left panel) [11]. When a preoperative ultrasound and an intraoperative frozen section do not reveal disease outside the thyroid gland, the proportion of sporadic tumors confined to the thyroid gland almost trebles to three-quarters (19 [76%] of 25 patients; Table 2, right panel) [14].

### 4.2. Interpretation

The current body of evidence confirms the viability of the novel diagnostic–therapeutic concept of frozen-section-guided hemithyroidectomy, complemented by a diagnostic ipsilateral central node dissection during the implementation phase, for one-third of patients with sporadic MTC. The reason for the addition of an ipsilateral central node dissection is to assure the operating team that the first stage of lymphatic drainage is free of tumors. This reassurance is helpful to minimize the risk of incorrect desmoplasia-negative findings by establishing a robust feedback loop to the frozen section unit at the operating surgeon’s institution, especially during the roll-out phase. With this strategy, only the undissected neck compartments need to be cleared in the exceptional instance of node recurrence.

This safeguard restricts the applicability of this innovative concept to suitably qualified surgical departments with integrated pathology units skilled in frozen section analysis.

Despite the less extensive surgery, patients with desmoplasia-negative solitary tumors on a frozen section have excellent postoperative clinical outcomes, with normal or absent calcitonin and CEA serum levels. In general, a hemithyroidectomy minimizes the need for postoperative thyroxine substitution, but a concomitant nodular thyroid disease, pervading both thyroid lobes, may require total thyroidectomy [14].

To optimize the surgical treatment for MTC, a preoperative ultrasound—complemented by an intraoperative frozen section—is the most important element in experienced hands for the early discrimination between encapsulated desmoplasia-negative and classic node-positive MTC [14].

**Figure 3:**
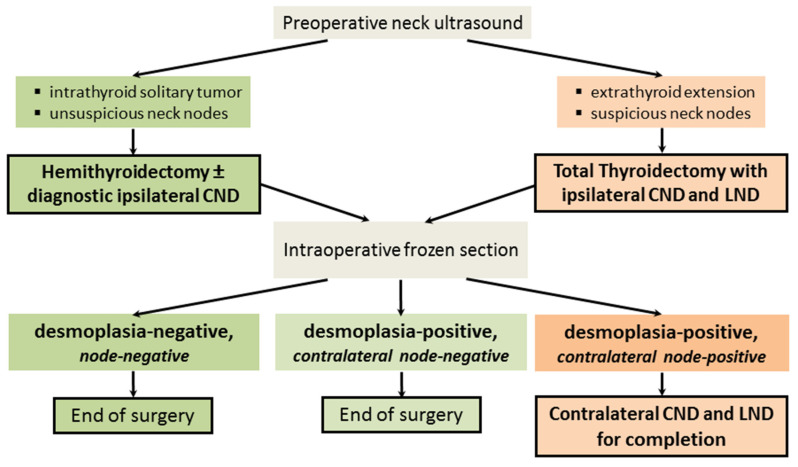
Risk-adapted surgical algorithm for sporadic medullary thyroid cancer. CND, central node dissection; LND, lateral node dissection. For desmoplasia-negative thyroid tumors, hemithyroidectomy alone is adequate therapy because these tumors are non-metastatic (Figure 3, green pathway; Figure 4A). The reason for combining a hemithyroidectomy with an ipsilateral central node dissection is to provide an extra safety margin into the risk-reducing concept.

For sporadic MTC, Figure 3 illustrates the novel risk-adapted surgical concept founded on a preoperative ultrasound and an intraoperative frozen section.

**Figure 4:**
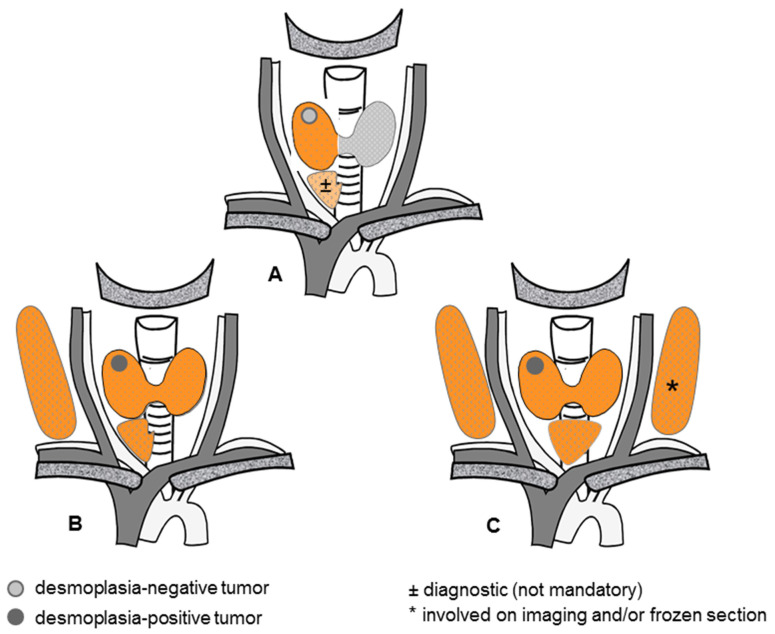
Risk-adapted surgical concept for sporadic medullary thyroid cancer. (**A**) Hemithyroidectomy, with or without a diagnostic ipsilateral central node dissection, for a desmoplasia-negative primary thyroid tumor, (**B**) Total thyroidectomy with concomitant ipsilateral central and lateral node dissections for a less advanced desmoplasia-positive primary thyroid tumor, (**C**) Total thyroidectomy with concomitant bilateral central and lateral node dissections for an advanced desmoplasia-positive primary thyroid tumor.

During the informed consent process, the importance of the intraoperative frozen section and the surgical consequences from the absence of primary tumor desmoplasia (Figure 4) should take center stage [14].

**Figure 5:**
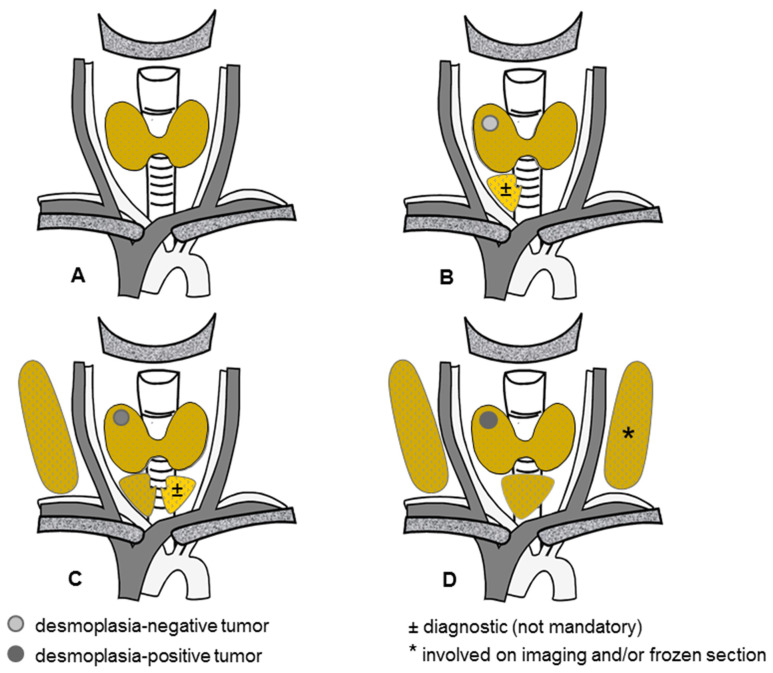
Emerging risk-adapted surgical concept for hereditary medullary thyroid cancer. (**A**) Total thyroidectomy, without node dissection, in the absence of an identifiable primary thyroid tumor, (**B**) Total thyroidectomy, with or without a diagnostic ipsilateral central node dissection, for one (or more) desmoplasia-negative primary thyroid tumor(s), (**C**) Total thyroidectomy with concomitant ipsilateral central and lateral node dissections, with or without a diagnostic contralateral central node dissection, for one (or more ipsilateral) less advanced desmoplasia-positive primary thyroid tumor(s), (**D**) Total thyroidectomy with concomitant bilateral central and lateral node dissections for one or more desmoplasia-positive primary thyroid tumor(s), one of which is advanced).

Hereditary MTC is a different issue. Because each thyroid C cell remaining behind carries its own risk of malignant progression, a total thyroidectomy is mandatory for any RET carriers (Figure 5).

Early preliminary evidence for multiple endocrine neoplasia 2B hints at the frequent co-existence of more than one primary tumor in the thyroid gland, each of which carries a distinct metastatic risk proportional to the degree of the primary tumor desmoplasia [24].

Equally large hereditary tumors and sporadic tumors appear to share the same biological behavior when the number of primary thyroid tumors is taken into account. If confirmed by larger independent series, the risk-adapted surgical concept for sporadic MTC (Figure 4) could be modified to accommodate multiple thyroid tumors dispersed throughout the thyroid lobes, which may be all desmoplasia-negative, desmoplasia-positive, or a mixture of both (Figure 5).

Because each C cell may give rise to MTC at some point, a total thyroidectomy represents the minimum treatment for hereditary MTC (Figure 5). Depending on the clinical constellation, a total thyroidectomy may need to be complemented by an ipsilateral (Figure 5B,C) and bilateral (Figure 5C,D) node dissection.

The rationale behind this suggestion is the strong link between desmoplasia-positive (but not desmoplasia-negative) MTC and ipsilateral node metastases.

Until more evidence is forthcoming, patients with hereditary MTC without evidence of neck node metastases by ultrasound examination and without evidence of distant metastases should continue to have a total thyroidectomy and central node dissection [32].

### 4.3. Generalizability

When a novel concept hits the operating floor, proficiency increases as experience grows. For safety reasons, it may be prudent to complement the hemithyroidectomy with an ipsilateral central node dissection, at least during the early phase of implementation. The adoption of the frozen-section-guided concept of risk-reducing surgery requires suitably qualified frozen section units within a short distance from the operating room, and the mastery of node dissection by the surgical team in the event of desmoplasia-positive MTC. These prerequisites limit the applicability of this risk-reducing surgery to high-volume tertiary centers for thyroid cancer with frozen section units.

### 4.4. Strengths and Limitations

The novel risk-reducing surgical concept enables the modification of surgical treatment plans guided by an intraoperative frozen section, helping to avoid reoperation for a persistent tumor. This key strength sets the risk-adapted surgical concept for MTC apart from conventional tumor classification systems [33,34], all of which rely on a postoperative definitive histopathology. Up to one-third of sporadic patients with desmoplasia-negative tumors on a frozen section can be spared additional morbidity by limiting the extent of the initial neck surgery, without compromising the cure.

Tumor heterogeneity may cause slightly deviating results in the histopathological interpretation of the primary thyroid tumors, depending on the area selected for immunostaining and analysis. Interestingly, Koperek et al. described an intra-observer concordance for desmoplasia on a definitive histopathology of 0.883, with inter-observer reliabilities ranging from 0.758 to 0.837 [22,23]. This observation favors the use of a binary grading scheme for primary tumor desmoplasia (absence vs. presence) in order to not increase the inter-observer variability [12,22,23].

## 5. Conclusions

For a desmoplasia-negative sporadic MTC, a hemithyroidectomy alone is an adequate oncological therapy. This desmoplasia-negative unifocal, non-metastatic neoplasm, also referred to as Sporadic Noninvasive Medullary Thyroid Neoplasm (SNIMTN) [14], ranges at the indolent end of medullary thyroid tumors, similar to noninvasive follicular thyroid neoplasm with papillary-like nuclear features (NIFTP) within the spectrum of follicular-cell-derived thyroid tumors [35]. Sparing the other thyroid lobe, when it is free of other thyroid disease, helps maintain sufficient thyroid hormone production, an ability which is lost with a total thyroidectomy.

The current knowledge gaps include the viability of a modified risk-reducing concept for hereditary MTC when preoperative calcitonin levels are borderline or mildly raised: a total thyroidectomy (to eliminate once and for all the risk of malignant progression) coupled with no or an ipsilateral (as opposed to bilateral) node dissection, if all the primary thyroid tumors have been shown to be node-negative on the frozen section.

Respect for patient autonomy, the right of competent adults to make informed decisions about their own medical care without their healthcare provider trying to influence their decision, and mandates the upgrading of organizational structures rather than withholding medical progress from eligible patients—all of these are reasons why more frozen section units should be set up in close proximity to suitably qualified surgical centers to make the benefits of the novel concept of risk-adapted surgery available to more patients with MTC.

## Figures and Tables

**Table 1:** Level of evidence, type of trial, criteria for classification, and grade of recommendation.

Level of Evidence [16]	Type of Trial [16]	Criteria for Classification [17]	Grade of Recommendation
I	Large randomized trials with clear-cut results (and low risk of error).	Sample size calculation provided and fulfilled, study endpoint provided.	A
II	Small randomized trials with uncertain results (and moderate-to-high risk of errors).	Matched analysis, sample size calculation not given or not fulfilled, study endpoints not provided, convincing comparative studies.	B
III	Nonrandomized, contemporaneous controls.	Noncomparative, prospective.	C
IV	Nonrandomized, historical controls.	Retrospective analysis, cohort studies.	–
V	No control, case series only; opinion of experts [17].	Small series, review articles.	–

**Table 2:** Characteristics of patients with desmoplasia-negative sporadic medullary thyroid cancer.

Reference		Scheuba et al. (2006) [11]			Dralle et al. (2023) [14]		
Primary Tumor Desmoplasia		Definitive Histology		*p*	Frozen Section *		*p*
		–	+		–	+	
No. of Patients		32	88		19	6	
Age at thyroidectomy, y		52.4 ± 11	62 ± 12	0.0003	54 [46; 66] (29–80)	54 [45; 72] (27–79)	0.985
Sex, no. of male patients		17 (53)	35 (40)	0.216	6 (32)	4 (67)	0.175
Preoperative basal calcitonin level, pg/mL		–	–		315 [125; 873] (34.3–49,167)	280 [194; 1327] (79.8–1406)	0.975
Preoperative carcinoembryonic antigen (CEA) level, ng/ml		6.2 ± 6.5 (*n* = 12)	85.9 ± 224 (*n* = 50)	0.08	20 [3; 54] (0.5–410) (*n* = 18)	10 [1; 21] (0.5–22.6)	0.140
No. of patients with	hemithyroidectomy	–	–	>0.999	12 (63)	2 (33) ^$^	0.350
	total thyroidectomy	32 (100)	88 (100)		7 (37) ^†^	4 (67)	
No. of patients with central neck dissection	none	0	0	>0.999	2 (11)	0 (0)	0.335
	ipsilateral	32 (100)	88 (100)		13 (68)	3 (50)	
	contralateral				4 (21)	3 (50)	
No. of patients with lateral neck dissection	ipsilateral	32 (100)	88 (100)	>0.999	2 (11)	5 (83)	0.002
	contralateral				0 (0)	0 (0)	>0.999
Primary tumor diameter, mm, median [IQR] (range)		–	–		17 [9; 25] (5–90)	15 [8; 20] (6–21)	0.427
No. of patients with multifocal growth		–	–		0 (0)	2 (33) ^‡^	0.050
No. of patients with extrathyroid extension		0 (0)	11 (14)	0.035	0 (0)	0 (0)	>0.999
No. of patients with node metastases		0 (0)	31 (35)	<0.001	0 (0)	6 (100)	<0.001
No. of node metastases removed		–	–		0 [0; 0] (0–0) (*n* = 17)	3.5 [3; 11] (1–14)	<0.001
No. of nodes removed		–	–		6 [4.5; 15.5] (1–25) (*n* = 17)	31 [22; 43] (20–53)	<0.001
No. of patients with biochemical cure		32 (100)	66 (75)	<0.001	19 (100)	5 (83)	0.240

The values in parentheses denote column percentages; the mean ± standard deviation or median [interquartile range]; —: data not provided; *: intrathyroid node-negative tumors on the preoperative ultrasound only; ^$^: on the patients’ request to avoid postoperative thyroxin replacement; ^†^: for concurrent bilateral nodular disease; ^‡^: limited to the same thyroid lobe.

## Data Availability

The data presented in this study are in the public domain and available from the international literature.
